# News and Miscellany

**Published:** 1903-04

**Authors:** 


### News and Miscellany.

FELLOWS: Don't forget to put an extra dollar in your pocket for the "Red Back," when you start to San Antonio.

---

THE BRAZOS VALLEY MEDICAL ASSOCIATION will meet at Cameron, Texas, May 12, prox. Dr. W. B. Briggs, Secretary, Easterly, Texas.

---

DR. J. T. HARRINGTON of Waco has been appointed by the Governor a member of the board of trustees of the Epileptic Asylum at Abilene.

---

THE NASHVILLE ACADEMY OF MEDICINE gave a great banquet at Tulano Hotel on April 7, inst., in honor of Dr. T. S. Madden, on the anniversary of his fiftieth year as a member of the Academy.

FOR SALE: Five room house, necessary out-buildings, for $600.00, half cash. Practice averages about $2500. Established eight years, no competition. Address at once,

T. L. TREADAWAY, M. D., Lilac, Texas.

---

THE STATE BOARD OF MEDICAL EXAMINERS (Regular) will meet in Austin, April 20th, inst. Examination of applicants for license to practice will be held on the first floor of the Driskill Hotel, beginning at 10 o'clock, a. m.

---

EXAMINATIONS of urine, sputum, blood, pathological specimens, etc., made at reasonable rates by New Orleans Clinical Laboratory, 124 Baronne Street, New Orleans. O. L. Pothier, M. D., Charity Hospital. I. I. Lemann, M. D., Secretary. J. B. Guthrie, M. D.

---

DR. W. R. BLAYLOCK, of McGregor, is at the New York Polyclinic. Dr. Blaylock will visit the great medical centers of England and Europe and return to Texas in the fall. We commend him to the profession everywhere as worthy of their friendship and esteem.

---

THE MEDICAL DEPARTMENT, TRINITY UNIVERSITY, the Dallas Medical College, held its commencement exercises in Carnegie Hall, Dallas, April 1. There were twenty-five graduates in medicine and three in pharmacy. Dr. H. L. McNew is Dean of the Faculty.

---

ELLA LOUISA ASTON is the name of an eleven pound and ten-horse-power-pretty daughter recently sent from Heaven to my young friends, Dr. and Mrs. S. Aston of Buffalo, Texas. Mrs. Aston is a daughter of the late Dr. A. D. Boggs and sister of Dr. E. Orleans Boggs of Marquez.

---

LOUISVILLE, KY., February 24, 1903.

*Publishers Texas Medical Journal, Austin, Texas.*

You may renew our ad. Long live the "Red Back" and its genial editor. ARTHUR PETER & Co.

(Eighteenth consecutive year.)

---

THE INTERURBAN ELECTRIC RAILWAY, Dallas and Fort Worth. We have received a beautifully illustrated pamphlet descriptive of this splendidly equipped line, and it is a revelation to us. We had no idea of the elegance and large proportions of this plant. Mr. W. C. Forbes, General Passenger and Freight Agent, Fort Worth, will be pleased to send a copy of the folder free on request.

---

NEW ORLEANS POLYCLINIC.—Sixteenth annual session opens November 3, 1902, and closes May 3, 1903. Physicians will find the Polyclinic an excellent means for posting themselves upon modern progress in all branches of medicine and surgery. The specialties are fully taught, including laboratory work. For further information, address New Orleans Polyclinic, Postoffice Box 797, New Orleans, La.

---

A STATE PHARMACEUTICAL BOARD OF EXAMINERS is provided for by Capt. Love's (Dallas) Pharmacy bill, which passed. It is very strict, and justly so. It requires the examination of all who compound or dispense drugs, and a license, to be registered. It carries no appropriation, but will be self-sustaining and perhaps a source of revenue to the State. The Governor may, very likely, veto it on account of certain objectionable features.

---

A pupil in a village school who had been requested to write an essay on the human body handed in the following: "The human body consists of the head, thorax, abdomen and legs. The head contains the brains in case there are any. The thorax contains the heart and lungs, also the liver and lights. The abdomen contains the bowels, of which there are five—a, e, i, o, u, and sometimes w and y. The legs extend from the abdomen to the floor, and have hinges at the top and middle to enable a fellow to sit when standing or to stand when sitting."

---

DON'T FORGET that the Texas State Medical Association will meet in San Antonio April 28 to May 1, inclusive. It will be the most important meeting the Association ever held. A new constitution is to be adopted; that is, the society is to be reorganized, and there is going to be a lively discussion over the plan that will be presented. The committee on State Board of Health will report, also, and as there is a division in the committee there will be an interesting discussion. San Antonio, lovely at all times, will be simply charming in her new Easter attire, flowers and soft breezes, lovely women and brave men. The people know, too, how to entertain the doctors, and an enjoyable time is assured. Be there, Doctor, without fail.

---

THE BILL by Dr. Miller, Representative from Milam, to prevent substitution on physicians' prescriptions, never saw daylight in either house. I was informed by Dr. Miller that he could never get recognized by the Speaker to bring it up. It was reported favorably by the Public Health committee, and recommended to pass. It is said that the reason the Speaker would not let the bill come up is because he was deluged with petitions against it from retail druggists in Houston, Beaumont and other cities where, doubtless, the nefarious practice of substitution is carried on. The medical profession should see to this. Every physician and his patients are "at the mercy" of these substituting devils. They should every one of them be in State prison.

---

AND I DON'T WANT you to forget, either, that the Great American Medical Association will meet in New Orleans, May 5 to 8, prox. This is the first time in years that the South has been favored with a meeting of the National Medical Association, and Southern doctors should avail themselves of the pleasures and benefits in store for them, and show their appreciation of the privilege by pouring out in large numbers to attend the meeting. We must elect a Southern man, too, to the presidency. A most attractive program will be presented, and the social features that will be provided will be varied and delightful. The committee of arrangements, of which Dr. Isadore Dyer is chairman, have spared no labor or expense in getting up a pleasing program. It will include an excursion to Havana. All the railroads will sell tickets away down, and hotels will knock off for the benefit of visiting physicians and their ladies. Turn out, fellows, and let's be there in strong force.

